# Assessing the application of adapted theory of planned behaviour in predicting postpartum family planning intentions in a pragmatic randomized control trial in Western Kenya

**DOI:** 10.1371/journal.pone.0315029

**Published:** 2025-02-05

**Authors:** Morris Senghor Shisanya, Mary Kipmerewo, Everlyne Morema, Collins Ouma

**Affiliations:** 1 Department of Community Health Nursing, School of Nursing, Kibabii University, Bungoma, Kenya; 2 Department of Reproductive Health, Midwifery and Child Health, School of Nursing, Midwifery and Paramedical Sciences (SONMAPS), Masinde Muliro University of Science and Technology (MMUST), Kakamega, Kenya; 3 Department of Biomedical Sciences and Technology, Maseno University, Maseno, Kenya; Tabriz University of Medical Sciences, ISLAMIC REPUBLIC OF IRAN

## Abstract

**Introduction:**

In developing countries like Kenya, addressing the high population growth rate necessitates a focus on early Postpartum Family Planning (PPFP) use. Despite the critical need for PPFP, few researchers explore the application of health behaviour change theories to enhance FP use among postpartum women. This study assesses the application of adapted Theory of Planned Behaviour (TPB) in predicting intention for early PPFP in postpartum women in Western Kenya.

**Methods:**

This randomized control trial included pregnant women aged 15 to 49 attending Antenatal Care (ANC) clinics, randomly assigned to the "Nurses’ arm," "Community arm," or "Control arm." The intervention provided family planning (FP) counseling. Trained nurses and Community Health Workers (CHW) delivered counseling in their respective arms, while the control arm received routine care. Adapted TPB was integrated into client exit interviews to identify constructs influencing early PPFP intentions. Structural equation modeling (SEM) was used to predict the intention for early PPFP in the adapted TPB.

**Results:**

The SEM was optimized with the removal of client knowledge on early PPFP. The final model retained satisfaction with PPFP counseling, perceived normative beliefs, attitude towards PPFP, behaviour control of PPFP choice, and perceived risk of early postpartum pregnancy. Only satisfaction with counseling (P = 0.001), perceived normative beliefs (P<0.0001), attitude towards PPFP (P<0.0001), and behaviour control of PPFP choice (P = 0.018) significantly influenced early PPFP intention.

**Conclusion:**

The study demonstrates a viable application of the adapted TPB model in predicting early PPFP intention in an interventional study.

**Trial registration:**

The study was registered by the Pan African Clinical Trial Registry on 03 July 2021 with a Trial Registration Number PACTR202107891858045. The trial was prospectively registered.

## Introduction

The Theory of Planned Behaviour (TPB) is a widely used psychological theory that was first proposed by Icek Ajzen in 1985. The theory has been commonly used to predict and explain health behaviour. The theory proposes that a person’s behaviour is determined by their intention to engage in that behaviour, which is influenced by their attitudes, subjective norms, and perceived behavioural control [1].

In the context of health behaviours, attitudes refer to a person’s positive or negative feelings about a behaviour that affects their health. The Theory of Planned Behaviour (TPB) proposes that a person’s attitude towards a behaviour is one of the predictors of intention to engage in that behaviour. Attitudes are formed based on a person’s beliefs about a behaviour and the evaluation of those beliefs. A person may have a positive attitude towards contraception if they believe that it is beneficial for their health, and negative if they believe that it is has far worse side effects than benefits or are not effective for their intended purpose of spacing or limiting births [2,3]. Attitudes are considered to be the most important predictor of a person’s intention to engage in a behaviour, as they reflect the degree of evaluation of the behaviour. Positive attitudes towards a behaviour are associated with a higher intention to engage in that behaviour, while negative attitudes are associated with a lower intention [4]. It is important to consider participant attitudes when designing interventions aimed at promoting healthy behaviours. Research has shown that attitude is a strong predictor of behaviour, and focus on changing attitudes is effective in promoting healthy behaviours [5].

The TPB also proposes that subjective norms, or the perceived social pressure to engage in a behaviour, as another key factor that influences a person’s intention to engage in a behaviour. Subjective norms are based on a person’s perception of the attitudes and behaviours of significant others, such as family, friends, and healthcare providers, towards a behaviour. As such, a person may be more likely to use contraception if they believe that their friends and family also use contraception, or if their healthcare provider encourages them to space their births or limit the number of children they bear for health and socioeconomic reasons [6,7]. When a person perceives that significant others approve of a behaviour, they are more likely to have a positive intention to engage in that behaviour, and vice versa [7]. Subjective norms are important to consider when designing interventions aimed at promoting healthy behaviours, such as use of contraception, healthy eating, and regular exercise. Intervention aimed at increasing FP uptake may involve encouraging social support for FP, through social networks being encouraged to use and give psychosocial support towards use of FP. Likewise, opinion leaders, community-owned resource persons, healthcare providers can be used as promoters of FP use [8].

Equally, perceived behavioural control, or the extent to which a person believes they have control over performing a behaviour, is one of the key factors that influence a person’s intention to engage in that behaviour. Perceived behavioural control is based on a person’s beliefs about the availability of resources and the ease or difficulty of performing the behaviour. A person may be more likely to use FP if they believe that they have access to facilities that offer a variety of FP commodities to choose from and that it is easy to access and use without much inconvenience [9]. Perceived behavioural control is a key determinant of behaviour, as a person’s perception of the ease or difficulty of performing a behaviour has a direct impact on their intention to engage in that behaviour. If a person believes that exercising regularly is easy and they have the resources to do so, they will be more likely to intend to exercise regularly, and vice versa. Perceived behavioural control is important to consider when designing interventions aimed at promoting healthy behaviours, such as FP, regular exercise, healthy eating, and medication adherence. Research has shown that interventions that focus on changing perceived behavioural control are effective in promoting healthy behaviours [5,10]. Therefore, perceived behavioural control plays a crucial role in TPB, as it reflects a person’s beliefs about their ability to perform a behaviour and it’s an important predictor of a person’s intention to engage in a behaviour [11,12].

On the other hand, intention refers to a person’s plan to engage in a behaviour that affects their health, such as smoking cessation, drug adherence, healthy eating, or regular exercise. The theory proposes that a person’s intention to engage in a behaviour is influenced by their attitudes, subjective norms, and perceived behavioural control. If a person has a positive attitude towards a behaviour, perceives social pressure to engage in that behaviour, and believes that they have control over performing the behaviour, their intention to engage in that behaviour will be strong [1]. In TPB, intention is the most proximal predictor of behaviour, as it reflects a person’s plan to engage in a behaviour. A strong intention to engage in a behaviour is associated with a higher likelihood of actually engaging in that behaviour, while a weak intention is associated with a lower likelihood [12,13]. Interventions aimed at promoting healthy behaviours should focus on increasing a person’s intention to engage in that behaviour. This can be done by changing attitudes, subjective norms, and perceived behavioural control [6].

Intention, therefore, plays a crucial role in the TPB as it reflects a person’s plan to engage in a behaviour and it is considered to be the most proximal predictor of behaviour. Interventions aimed at promoting healthy behaviours should focus on increasing a person’s intention to engage in that behaviour by changing attitudes, subjective norms, and perceived behavioural control. So far, TPB has been widely used to predict and explain health behaviours and has been supported by numerous studies in various fields such as physical activity, diet, and medication adherence [2]. The theory can be used to design interventions that aim to increase the likelihood of a desired health behaviour by changing attitudes, subjective norms, and perceived behavioural control [10].

### Justification of the model

The efficacy of the TPB in predicting individual health-related behaviours has been demonstrated in several systematic reviews. Systematic review focused on the relationship between intention and behaviour have predicted more than 20% variance in prospective measures of the actual behaviour of individuals. This variance in behaviour as explained by intention is similar in magnitude to that found in the extant literature [11]. Since the TPB has been useful in predicting health-related behaviour, it may also be useful in evaluating behaviour among postnatal mothers’ fertility intentions and choices [3,13].

Application of the TPB in any new context requires a tool to measure the variables related to the behaviour of interest and its correlations and, like any other measurement tool, it should demonstrate evidence of psychometric properties, such as validity and reliability. There is adequate evidence that supports the successful use of TPB to predict the PPFP use. Therefore, there are measurement instruments whose reliability and validity is known with high internal consistency and test-retest reliability to predict fertility intentions and PPFP choice [1,11,14,15].

**Fig 1:**
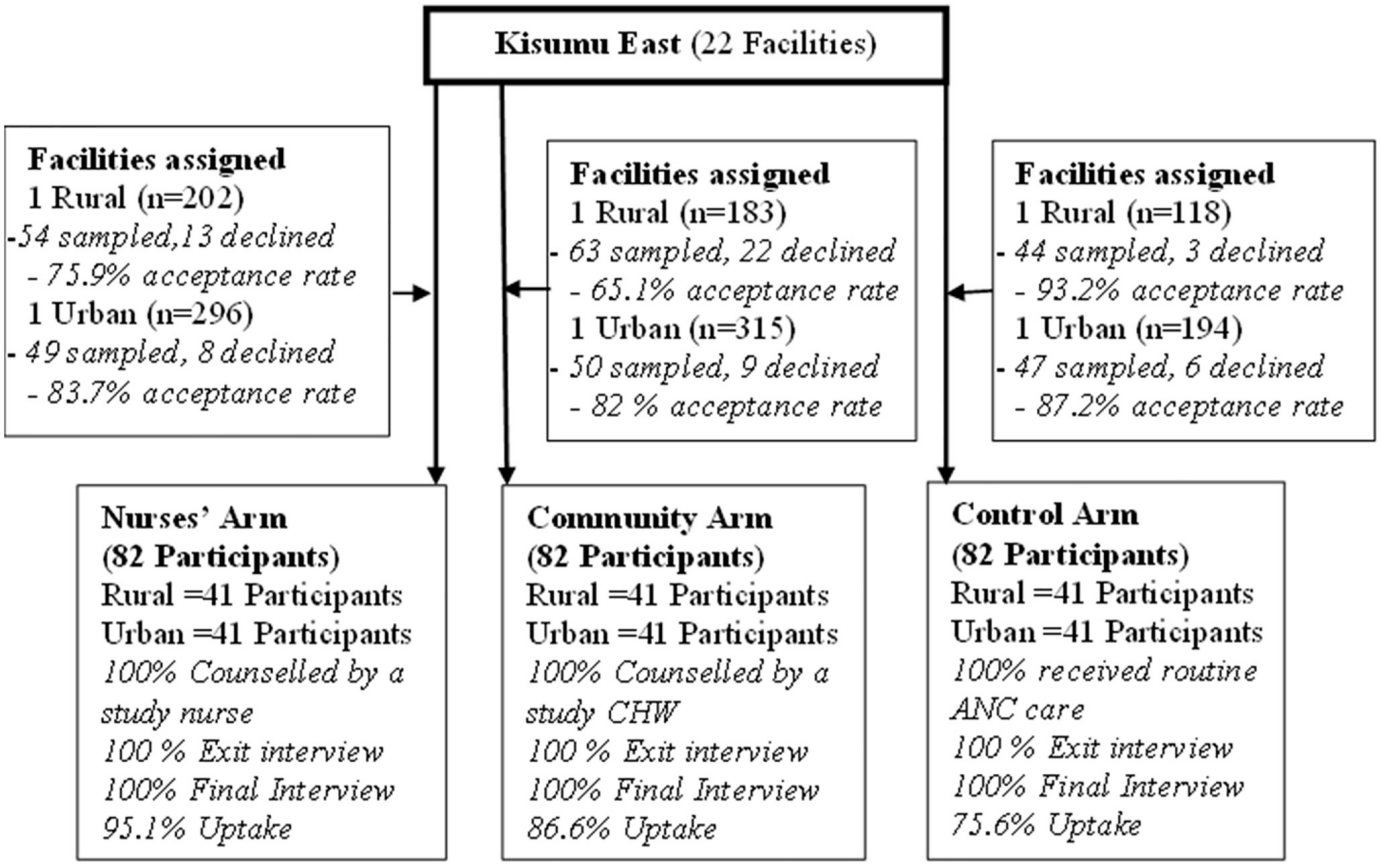
CONSORT diagram showing participant flow.

Lastly, it is true that the TPB is focused on the controlled facets of human judgment, intention and decision-making. According to the theory, subjective information in the form of behavioural, normative, and control beliefs provides the foundation for attitudes, intention and ultimately behaviour. This being a cluster randomized control trial, TPB was best placed to estimate fertility intention and eventually PPFP choices [1]. Thus, the study sought to assess the application of adapted Theory of Planned Behaviour (TPB) in predicting intention for early PPFP in postpartum women in Western Kenya. The study comprised three phases: pre-intervention, intervention, and follow-up as shown in Fig 1.

### Conceptualization of theoretical framework

In the current study, TPB was conceptualized into a framework that was used to explain the interaction of variables in determining intention and choice for PPFP. The framework offers means to study the interactions different aspects that determine fertility intentions, choice and usage of PPFP methods.

As much as TPB is a widely validated and used framework for understanding and predicting human behaviour, researchers have recognized that there are other factors that may influence behaviour, and the TPB can be modified by adding these constructs to the model [16]. Other exogenous constructs such as knowledge of behaviour, perceived risk of contrary behaviour, and satisfaction with behaviour-enhancing process can be adopted to TPB to optimize it. These constructs have previously been found to be important predictors of behaviour in various studies and can provide additional insight into the factors that influence intention and behaviour [10,17].

Research has shown that knowledge of behaviour can increase the accuracy of predictions made by TPB, as individuals with more knowledge about a behaviour may be more likely to engage in it. Knowledge of behaviour is an important predictor of behaviour because it can influence an individual’s attitudes and beliefs about the behaviour, as well as their ability to perform the behaviour. Research has shown that increasing knowledge about a particular behaviour can lead to more positive attitudes towards the behaviour and greater perceived behavioural control, which in turn, can increase the likelihood of engaging in the behaviour [6,18]. A previous study found that individuals who had more knowledge about the benefits of regular physical activity were more likely to engage in regular physical activity. The study found that increasing knowledge about the benefits of physical activity increased positive attitudes towards physical activity, which led to greater intentions to engage in physical activity [4]. Another study found that individuals who had more knowledge about the benefits of recycling were more likely to recycle. The study found that increasing knowledge about recycling led to more positive attitudes towards recycling, which in turn, led to greater intentions to recycle. Adding a construct of knowledge of behaviour to the TPB can increase the accuracy of predictions made by the theory because knowledge has a role in shaping attitudes and perceived behavioural control [19].

Perceived risk of not engaging in a desired behaviour, also known as "perceived risk of contrary behaviour," can be added as an adopted construct in the TPB model to understand why individuals may engage in a behaviour after considering the potential risk of not engaging in the said behaviour. For example, an individual may exercise regularly because they perceive risks of not exercising, such as potential negative health outcomes or social disapproval. They may believe that if they don’t exercise regularly, they will gain weight, have high blood pressure or become less attractive to others. In this case, the perceived risk of not exercising would be high and would likely encourage the individual to engage in regular physical activity [20,21]. Another example would be an individual who is considering quitting smoking. They may consider quitting because they may perceive the risks of not quitting (contrary behaviour), such as potential weight gain, stress, or social isolation. Research has shown that the perceived risk of not engaging in a behaviour can be a significant barrier to behaviour change. A study found that perceived risk of not engaging in a behaviour was a strong predictor of intentions [5].

Satisfaction with the process towards behaviour refers to an individual’s level of satisfaction with the steps or actions they take to engage in a behaviour. It can be an important predictor of behaviour because it can influence an individual’s motivation to continue engaging in a behaviour over time. An individual may have positive attitudes towards regular contraceptive use, perceive social pressure to be active, and have the ability to be active, but if they are not satisfied with the process of finding a facility with their preferred method, they may not continue to engage in FP. In this case, their satisfaction with the process towards the behaviour of FP would be low and would likely discourage the individual from continuing to engage in the behaviour [17,22]. Including satisfaction with the process towards behaviour in TPB can help to understand why individuals continue to engage in a behaviour or not, even if they have positive attitudes towards it, perceived social pressure to do so and the ability to perform the behaviour.

Including these constructs in the TPB can help to increase the explanatory power of the theory and provide a more comprehensive understanding of how individuals make decisions about behaviour. A previous researcher found that when perceived behavioural control, subjective norms, and attitudes were combined with satisfaction with the process, the explanation of intentions and behaviour improved significantly [7].

Therefore, an individual’s intention to use contraceptives may be influenced by their attitudes towards contraceptive use (e.g. believing it is important for family planning), their perceptions of social pressure to use contraceptives (e.g. believing that their partner and friends use contraceptives), and their beliefs about the ease or difficulty of obtaining and using contraceptives (e.g. access to contraceptives and knowledge about different methods).

Nonetheless, researchers have recognized that there are other factors that may influence contraceptive use, and the TPB can be modified by adding these constructs to the model. Knowledge of behaviour is an important predictor of contraceptive use. Individuals who have more knowledge about different methods of contraceptives and their effectiveness may be more likely to use contraceptives because they understand the outcomes associated with them and may also have more skills to obtain and use them [23].

Perceived risk of contrary behaviour can be added as an additional construct in TPB model. Perceived risk of not using contraceptives refers to an individual’s assessment of the likelihood and potential consequences of not using contraceptives, such as unintended pregnancy or sexually transmitted infections [24].

**Fig 2:**
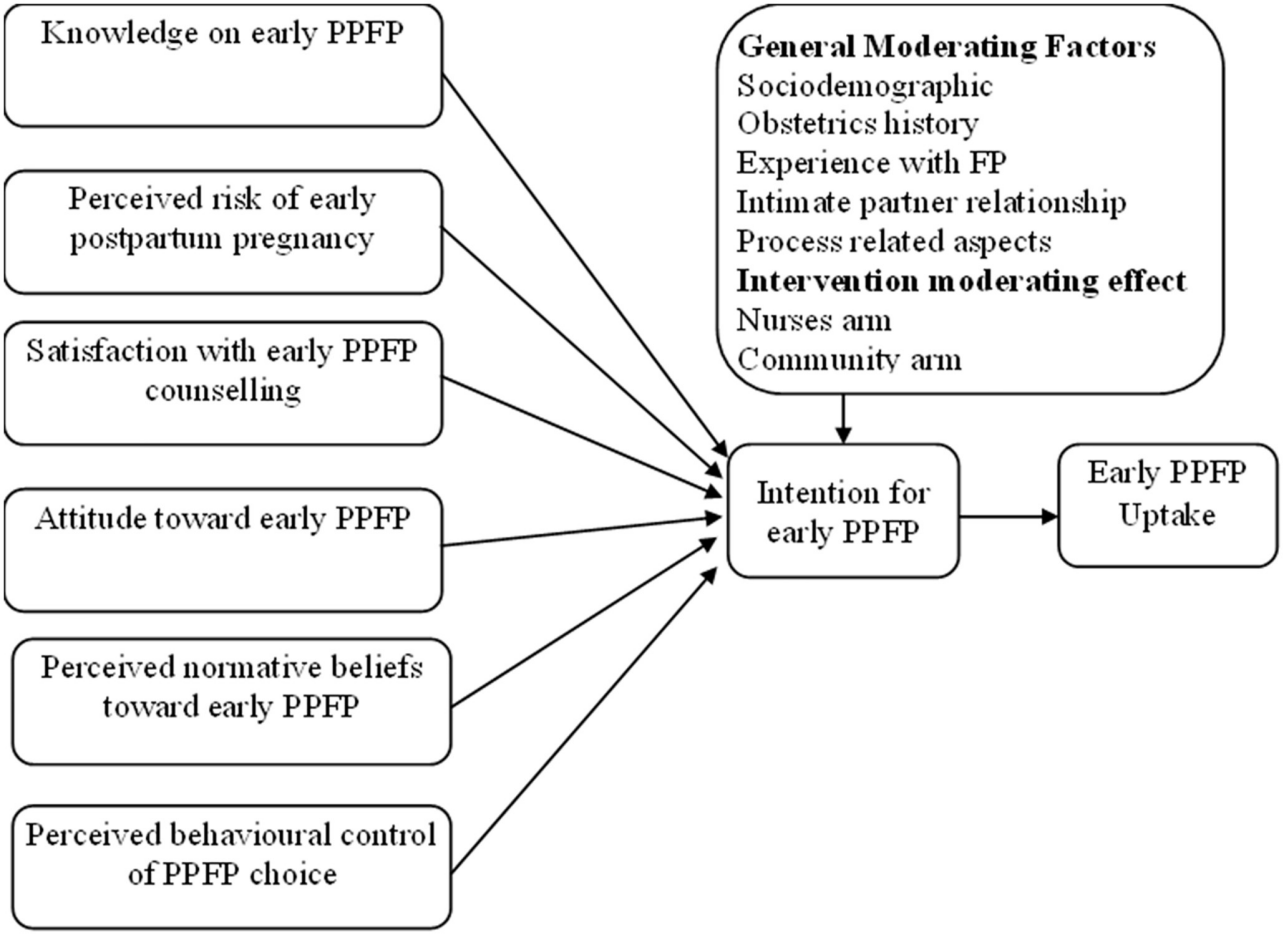
Conceptual framework constructs in the adapted TPB.

Satisfaction with the process towards behaviour is also an important predictor of contraceptive use. Individuals who are satisfied with the process towards obtaining and using contraceptives, such as access to information and services, may be more likely to use contraceptives and continue to use them over time. Studies have found that TPB can be used to predict contraceptive use by including satisfaction with the process to the core TPB constructs [15,25]. Fig 2 represents the conceptualized TPB applied to the current study with Table 1 providing the conceptual and operational definition of the constructs in the adapted TPB.

**Table 1:** Definition of concepts in the conceptual framework.

CONCEPT	DEFINITION	OPERATIONAL DEFINITION	STUDY VARIABLES
**Knowledge related to behaviour**	Person’s understanding of the behaviour, how and why it happens and when it’s appropriate to engage.	Knowledge of early PPFP.	Assessed by 5 parameters based on FP choice, benefits, side effects, risk-benefit analysis and knowledge on one of the methods.
**Perceived risk related to behaviour or contrary behaviour**	Individual’s assessment of the likelihood and potential consequences of engaging or not engaging in a particular behaviour.	Perceived risk of getting pregnant in early postpartum period.	Assessed by two parameters, perceived general risk and perceived individual risk of pregnancy in early postpartum period.
**Satisfaction with process towards behaviour**	Individual’s level of contentment with the steps taken to achieve a behaviour or goal.	Satisfaction with early PPFP counseling.	Satisfaction with the; information given on FP methods, Choices of PPFP available, Response to your questions and concerns, privacy, Respect to your opinion, Repetition of important points.
**Attitudes towards behaviour**	Psychological tendency that is expressed by evaluating target behaviour with some degree of approval or disapproval.	Attitudes towards early PPFP uptake.	Attitude towards uptake, attitude towards recommended minimum of 24 months of interbirth spacing, role of early PPFP in attaining recommended interbirth spacing and side effects of PPFP.
**Perceived normative belief**	Individual’s beliefs about what is considered normal or acceptable within their social or cultural group.	Perceived normative belief towards early PPFP.	Consisted of; whether significant others think that client should use PPFP, whether it is socially expected of client to use FP soon after delivery, whether there is social pressure to use FP soon after delivery, and whether the partner approves use of FP and Partner is open on FP talk.
**Perceived behavioural control**	Refers to an individual’s belief that they have the power to make decisions and influence events in their lives.	Perceived behavioural control of early PPFP choice.	Client’s confidence in using PPFP if she wants to, belief on ease of using PPFP and client’s control of the decision to use PPFP.
**Intention of early PPFP use**	Refers to an individual’s desire or determination to engage in a particular behaviour.	Indications of mother’s readiness to use PPFP.	Client; expects to use FP within first 3 months of delivery, wants to use family planning within the first 3 months of delivery and intends to use family planning within first 3 months of delivery.
**Behaviour**	An individual’s observable response in a given situation with respect to a given target.	Mother’s ability to use modern available PPFP methods during the first one year.	Commencement of PPFP within 3 months postpartum.

## Methodology

### Study design

This was a non-blinded pragmatic randomized controlled trial with three arms (nurses’ arm community arm, and control arm) and three phases (pre-intervention, intervention, and post-intervention) aimed at assessing the application of adapted TPB in predicting intention for early PPFP in postpartum women in Western Kenya.

### Study setting and procedure

The study focused on pregnant mothers in their second or third trimester attending ANC clinics in Kisumu County, Kenya. A total of 6 facilities were included in the study, with 2 facilities randomly assigned to each of the 3 arms (nurses’ arm, community arm, and control arm), including 1 rural and 1 urban facility for each arm.

The sample size was estimated based on a proposed sample size determination formula for difference in proportions with consideration of type I and II errors and power [26–30], to be: N1={z_(1−α/2)*√(p¯*q¯*(1+1/k))+z_1−β*√(p1*q1+(p2*q2)/k)}^2/Δ^2. Where q1 = 1-p1, q2 = 1-p2, $\bar{\text{p}}=\left(\text{p}1+\text{kp}2\right)/\left(1+\text{K}\right)$, p1, p2 = proportion (incidence) of groups #1 (27% which is the KDHS estimated current PPFP use) and #2 (53% which is the KDHS estimated Contraceptive Prevalence Rate (CPR) in the general population), Δ = |p2-p1| = absolute difference (desired clinical difference between intervention and control arms) between two proportions (0.26 i.e. 0.53–0.27), n1 = sample size for group #1, n2 = sample size for group #2, α = probability of type I error (is set at 0.05), β = probability of type II error (is set at 0.1 i.e. 90% power), z = critical Z value for a given α or β(1.96) and K = ratio of sample size for group #2 to group #1(1). Thus, for practical equal sample distribution with assumed 10% loss to follow-up, the actual sample size was 246 with each study arm having 82 participants. As such, each facility per arm based on the rural-urban dichotomy had 41 participants [31].

The Health Centres were eligible to participate in the study if they met certain criteria such as offering full ANC, delivery, and PNC services, providing at least three modern contraceptive methods, having no recent contraceptive stock-outs, performing at least 10 deliveries per month, and being willing to participate. Pregnant women in their second or third trimester, attending ANC, intending to attend PNC at the Health Centre, providing informed consent, and living within 20km were eligible to participate. Excluded from the study were; participation in another study, latex sensitivity, lack of a male partner in the next 12 months, inability to complete the consent form, or the only male partner having a vasectomy [32].

The study used a multistage sampling method that combined purposive sampling for the sub-counties and facilities and simple random sampling for the subjects. Participants were randomly assigned to the study through simple random sampling using excel random numbers applied to the sampling frame provided in each facility as per those who met the eligibility criteria. Randomization was done by the principal investigator.

The study comprised three phases: pre-intervention, intervention, and follow-up. During the pre-intervention phase which lasted approximately 6 months from July 2021, site selection and preparation were carried out. Study preparation involved research staff training, tool preparation, tool piloting, finalization, publication of study protocols, and preregistration of protocols with a recognized clinical trials registry (PACTR). The intervention phase encompassed recruitment, random allocation of participants, and administering a single session of antenatal early postpartum family planning (PPFP) information provision in the third trimester. The intervention phase of the study lasted from 26^th^ February to 30^th^ August 2022. It involved counseling on early PPFP delivered by trained nurses and community health workers. The study followed clients to assess the quality of the counseling session and determine their intention to use early PPFP. The follow-up phase occurred 14 and 20 weeks post-delivery to assess PPFP uptake in the first three months after delivery, commencing 23^rd^ May 2022 to 10^th^ Feb 2023. The during the study, there was continuous monitoring and necessary corrective actions were taken. Clients who lost their sexual partner, underwent postpartum psychosis, or were hospitalized for more than 14 weeks postpartum were discontinued from the study.

The study trained research assistants at each Health Centre and community unit to ensure data management compliance. The primary outcomes were the intention and actual use of early PPFP. The ANC service provider filled out the Case Report Forms at recruitment, and the counselor filled out an appointment card after the client agreed to a follow-up date for PPFP. The client’s exit interview was done after the intervention to assess process quality and measure TPB constructs parameters.

### Measurement

Adapted TPB model constructs were knowledge PPFP, satisfaction PPFP counseling, perceived normative beliefs on early PPFP, attitude towards early PPFP, behaviour control of PPFP choice, PPFP intention, perceived risk of early postpartum pregnancy, and uptake of PPFP in the early postpartum period.

The core concepts were measured using adapted validated tool as per the manual for studying TPB by Francis et al., 2004. The primary outcome which was the modeled behaviour was uptake of PPFP in the early postpartum period at 3 months post-delivery. Five tools were used for data collection, namely; client exit interview guide, case report form, appointment card, Site appraisal form and questionnaire. All the tools were used to collect quantitative data except site appraisal form and some questions in the questionnaire that need brief explanation.

The Theory of Planned Behaviour was applied to design the tools. Phone based (Kobo Collect) Case Report Forms (CRFs) was used in recruitment of clients to the study. These CRFs had study’s eligibility criteria, client’s biodata, past and current obstetric information of the client, and past and present medical history of the of client.

Client exit interview guide and Site appraisal form were developed based on the procedures set out in the counseling guide and the overall set up of the counseling session. Appointment card was source of information on client details, proposed date for PPFP initiation and vital PPFP information summary. The other quantitative data collection tool was questionnaire during postpartum follow up. The questionnaire was tailored to the recommendations of Francis et al., 2004 and Ajzen and Klobas 2013 [1,33]. The tools were piloted in advance with an appropriate sample of service providers, CHWs and mock clients from the pilot sites before finalizing the forms.

Attitude towards early PPFP was directly measured using four 7-point Likert scale (Cronbach’s alpha 0.844) ranging from strongly agree (7) to strongly disagree (1). Attitude was then summarized into a continuous variable by calculating the mean score of parameter Likert scores. The mean scores were scaled to classify attitude as follows; very negative attitude (≥1, <2); moderate negative attitude (≥2, <3); weak negative attitude (≥3, <4); neutral attitude (≥4, <5); weak positive attitude (≥5, <6); moderate positive attitude (≥6, <7) and very positive attitude (≤7) [33].

Perceived normative beliefs on early PPFP was directly measured using five (5) 7-point Likert scale paired variables (Cronbach’s alpha 0.807) ranging from strongly agree (7) to strongly disagree (1). Perceived normative beliefs was then summarized into a continuous variable by calculating the mean score of parameter Likert scores. The mean scores were scaled to classify perceived normative beliefs as follows; very positive (7), positive (≥6 <7), moderately positive (≥5 <6), neither positive nor negative (≥4 <5), moderately negative (≥3 <4), negative (≥2 <3), and very negative (≥ <2).

Behavioural control was measured by three (3) 7-point Likert scale questions (Cronbach’s alpha 0.779) ranging from strongly agree (7) to strongly disagree (1). Behavioural control was then summarized into a continuous variable by calculating the mean score of parameter Likert scores. The mean scores were scaled to classify perceived behavioural control as follows; very high (7), high (≥6 <7), moderately high (≥5 <6), neither high nor low (≥4 <5), moderately low (≥3 <4), low (≥2 <3), and very low (≥1 <2).

Intention to use early PPFP was directly determined by three (3) 7-point Likert scale questions (Cronbach’s alpha 0.850) ranging from strongly agree (7) to strongly disagree (1). Intention was then summarized into a continuous variable by calculating the mean score of parameter Likert scores. The mean scores were scaled to classify intention as follows; very high intention (= 7), high intention (≥6, <7), moderately high intention (≥5, <6), neither high nor low high intention (≥4, <5), moderately low intention (≥3, <4), low intention (≥2, <3), and very low intention (≥1, <2).

The constructs adapted into the TPB model were knowledge of PPFP, satisfaction with PPFP counseling, and perceive risk of early postpartum pregnancy. Knowledge of PPFP was directly determined by six (6) 7-point Likert scale questions (Cronbach’s alpha 0.688) ranging from strongly agree (7) to strongly disagree (1). Knowledge was then summarized into a continuous variable by calculating the mean score of parameter Likert scores. The mean scores were scaled to classify knowledge as follows; very high knowledge (= 7), high knowledge (≥6, <7), moderately high knowledge (≥5, <6), neither high nor low high knowledge (≥4, <5), moderately low knowledge (≥3, <4), low knowledge (≥2, <3), and very low knowledge (≥1, <2).

Satisfaction with PPFP counseling was directly determined by six (6) 7-point Likert scale questions (Cronbach’s alpha 0.892) ranging from very satisfied (7) to very dissatisfied (1). Satisfaction was then summarized into a continuous variable by calculating the mean score of parameter Likert scores. The mean scores were scaled to classify satisfaction as follows; very satisfied (7), satisfied (≥6, <7), moderately satisfied (≥5, <6), neither dissatisfied nor satisfied (≥4, <5), moderately dissatisfied (≥3, <4), dissatisfied (≥2, <3), and strongly dissatisfied (≥1, <2).

Perceive risk of early postpartum pregnancy was directly determined by two (2) 7-point Likert scale questions (Cronbach’s alpha 0.823) ranging from very satisfied (7) to very dissatisfied (1). Perceive risk of early postpartum pregnancy was then summarized into a continuous variable by calculating the mean score of parameter Likert scores. The mean scores were scaled to classify Perceive risk of early postpartum pregnancy as follows; very high perceived risk (= 7), high perceived risk (≥6, <7), moderately high perceived risk (≥5, <6), neither high nor low high perceived risk (≥4, <5), moderately low perceived risk (≥3, <4), low perceived risk (≥2, <3), and very low perceived risk (≥1, <2).

### Data analysis

In TPB, intention is an immediate determinant of behaviour thus the effect of the other constructs; Knowledge of PPFP, satisfaction with counseling process, perceived risk of getting pregnant in the early postpartum period, individual attitude towards behaviour, perceived normative beliefs about the behaviour, and perceived individual control of the behaviour are mediated by intention. Therefore, Structural Equation Modeling (SEM) was done using SPSS AMOS version 21 to assess and propose modifications to the model for improvement of model fit indices. Factor analysis of the parameters and complete path analysis for the constructs in TPB was performed with adjustment for model fit [34]. The exogenous variables were attitude towards PPFP in early postpartum period, perceived normative beliefs towards PPFP in early postpartum period, behavioural control of choice of PPFP in early postpartum period, knowledge about PPFP in early postpartum period, satisfaction with counseling on PPFP in early postpartum period, and perceived risk of getting pregnant in the early postpartum period. The endogenous variable was intention. Intention was theorized as the direct determinant of behaviour was the last endogenous variable in the model. Five steps were inherent in the process of SEM; conceptualizing the model, constructing a measurement model in SPSS AMOS for Confirmatory Factor Analysis (CFA) and optimization of the model for fit, constructing a structural model using optimized CFA, full structural model path analysis and adopting a final model [35].

Reflective formative assessment of the measurement model was done with CFA. Factor loadings, correlation of constructs and their indicators, model fit indices and model modification suggestions generated by the software guided the process optimizing model fit so that its predictions could be generalizable. The model-fit indices were used to assess the model’s overall goodness of fit. Goodness of fit was assessed based on the prescribed thresholds for the C-Minimum Discrepancy Function by Degrees of Freedom (CMIN/df), Goodness of Fit Indices (GFI); the Tucker and Lewis (1973) Index (TLI); the Confirmatory Fit Index (CFI) [36,37]. Further assessment of fit was based on SPSS AMOS computed value of the Standardized Root Mean Square Residual (SRMR) and the Root Mean Square Error Approximation (RMSEA) [37,38]. The thresholds for the critical model indices were CMIN/df ≤3, GFI >0.9, CFI>0.9, TLI>0.9, SRMR<0.08 and RMSEA<0.08). Parameter’s reliability was assessed using Cronbach’s alpha >0.7 and composite R >0.7 for construct reliability. Convergent validity of scale items was estimated using Average Variance Extracted (AVE) >0.50 [39,40]. All P<0.05 were considered statistically significant.

Moderation effect of sociodemographic factors, process factors and quality of counseling, intimate partner relationship on the optimized model was assessed using ordinal regression analysis. The sociodemographic aspects assessed were age, residence, education level, income, and employment status. Residence, education level and employment status were converted to ranked order based on the trends established in the KDHS 2014 [41]. There was an increase in utilization of FP as age, level of education, income quantiles increased and those who were employed and those who lived in urban areas had higher FP utilization rates as compared to their counterparts. Therefore, marital status was assigned ranks as follows; never married “1”, separated “2” and married “3”. Level of education was assigned ranks as; no education and primary level education “1”, secondary education “2” and tertiary level education “3”. Employment status was assigned as follows; not employed “1”, housewife “2” self-employed “3” and formally employed “4”. Quality of counseling was based on 7-point Likert scale self-score on counseling process fidelity and client FP knowledge. Process factors were; staff score on counseling refresher training posttest, counseling waiting time and counseling turnaround time. Intimate partner relationship rating was assessed by Likert scale scoring for physical, sexual violence, and partner support willingness to be involved in FP decisions-making.

### Ethical consideration

The study was approved by Masinde Muliro University of Science and Technology (MMUST) School of Graduate Studies (SGS) (Ref: MMU/COR:509099). Ethical clearance was obtained from the MMUST Institutional Ethics Review Committee (IERC) (MMUST/IERC/013/2021). A research authorization permit was acquired from NACOSTI (Ref. No. 522628). An official data collection permission letter was obtained from the County Director of Medical Services (DMS). The trial was prospectively registered with the Pan African Clinical Trial Registry (PACTR), PACTR202109586388973. Signed written informed consent was obtained from all participants after they were introduced to the purpose of the study and informed about their rights. Refresher training was conducted for the nurses offering antenatal care services to the clients in both the intervention and control arms in order to maximize beneficence.

## Results

### Participant characteristics

The sample size was 246 pregnant women attending ANC were included in the study with each arm attaining 82 (100%) of the sample size. Participants had an age range of 16–42 years (M = 25.2, SD = 4.9) modal age group being 15–24 years. Most (84.1%) of the participants were married. The highest attained level of education for the participants was tertiary and more than 63% of the participants had attained high school or tertiary education. More than 86% of the participants and almost all the participants earned less than 5000 KES and were Christians, respectively. Mean age was 25.2 years (SD 4.9) was grouped into 3 groups with intra-cluster range of 10 years with a minimum age of 16 years and a maximum of 42 years. The modal age group was 15–24 years.

#### Early PPFP use

Early PPFP use was high at 85.8%. Study arms in the rural facilities realized higher PPFP commencement rates (90.2%) compared to the urban setting arm (81.3%). Table 2 shows that intervention arm had higher PPFP commencement rates (90.9%) than the control group (75.6%) (OR:3.2; 95% CI:1.5–6.7; P<0.0001). Nurses’ arm showed higher rates of commencement (95.1%) as compared to control OR:6.3; 95% CI: 2.0–19.4; P<0.0001 arm. Equally, community arm had higher numbers (86.6%) of participants starting PPFP than control arm (75.6%). The most common type of contraception was hormonal methods as follows; Implant (36.0%), injection DMPA (19.0%), and oral pills (16.1%). Barrier methods (9.5%) came in second after the hormonal methods and IUDs was third (8.1%). The rest of the methods accounted for 11.4% of the uptake.

**Table 2:** Commencement rates for early PPFP.

Study arm	Commenced FP		OR	95% CI	P Value	Effect size
	Yes N(%)	No N(%)				
All arms	211(85.8)	35 (14.2)				
Rural	111 (90.2)	12 (9.8)	**2.1**	**1.0–4.5**	**0.045**	0.13
Urban	100 (81.3)	23 (18.7)				
Intervention	149 (90.9)	15 (9.1)	**3.2**	**1.5–6.7**	**<0.0001**	0.21
Control	62 (75.6)	20 (24.4)				
Nurses’	78 (95.1)	4 (4.9)	**6.3**	**2.0–19.4**	**<0.0001**	0.28
Control	62 (75.6)	20 (24.4)				
Nurses’	78 (95.1)	4 (4.9)	3.0	0.9–9.9	0.051*	0.15
Community	71 (86.6)	11 (13.4)				
Community	71 (86.6)	11 (13.4)	2.1	0.9–4.7	0.073	0.14
Control	62 (75.6)	20 (24.4)				

This was a cross-tabulation of main study characteristics and commencement of PPFP. Effect size estimated by the Phi & Cramer’s V symmetry measure (0- no relationship, <0.2 weak, 0.2–0.3 moderate and >0.3 strong.

*Fisher’s exact test used for interpretation otherwise Pearson Chi-square was used.

### Measurement of TPB constructs

The adapted TPB model constructs were analyzed for basic descriptive statistics that summarized parameter Likert scale scores as shown in Table 3. Knowledge of early PPFP was assessed by 5 parameters based on FP choice, benefits, side effects, risk-benefit analysis and knowledge on one of the methods. Participant sentiments on each of the parameter were rated on 7-point Likert scale. The score was then averaged to get the overall knowledge score. The overall knowledge score was then re-rated to Likert score to express knowledge in an ordinal scale ranging from very low knowledge (1) to very high knowledge (7). The average score for knowledge was high 6.3 (SD = 0.5). Eighty-five percent (85.0%) of the participants’ knowledge rating of at least 6 (High). None of the participants rated below 4.

**Table 3:** Summary of TPB constructs parameter attributes distribution per study arm.

TPB Constructs	Summary of Parameter attributes	Study Arm			Total
		Nurses N(%)	Community N(%)	Control N(%)	
Knowledge level	Very high knowledge	24(80.0)	3(10.0)	3(10.0)	30(12.2)
	High knowledge	53(29.6)	71(39.7)	55(30.7)	179(72.8)
	Moderately high knowledge	5(14.3)	8(22.9)	22(62.9)	35(14.2)
	Neither high nor low knowledge	0(0.0)	0(0.0)	2(100.0)	2(0.8)
	**Total**	**82(33.3)**	**82(33.3)**	**82(33.3)**	**246(100)**
Perceived Risk	Very High Perceived Risk	17(41.5)	10(24.4)	14(34.1)	41(16.7)
	High Perceived Risk	59(34.7)	57(33.5)	54(31.8)	170(69.1)
	Moderately High Perceived Risk	4(33.3)	4(33.3)	4(33.3)	12(4.9)
	Neither High nor Low High Perceived Risk	0(0.0)	3(75.0)	1(25.0)	4(1.6)
	Moderately Low Perceived Risk	0(0.0)	2(28.6)	5(71.4)	7(2.8)
	Low Perceived Risk	2(16.7)	6(50.0)	4(33.3)	12(4.9)
	**Total**	**82(33.3)**	**82(33.3)**	**82(33.3)**	**246(100)**
Satisfaction with PPFP Counseling	Very satisfied (7)	47(61.8)	15(19.7)	14(18.4)	76(30.9)
	Satisfied (> = 6 <7)	35(21.5)	61(37.4)	67(41.1)	163(66.3)
	Moderately Satisfied (> = 5 <6)	0(0.0)	6(85.7)	1(14.3)	7(2.8)
	**Total**	**82(33.3)**	**82(33.3)**	**82(33.3)**	**246(100.0)**
Attitude	Very Positive Attitude (= 7)	9(36.0)	9(36.0)	7(28.0)	25(10.2)
	Moderate Positive Attitude (= 6, <7)	58(39.5)	35(23.8)	54(36.7)	147(59.8)
	Weak Positive Attitude (= 5, <6)	15(23.1)	32(49.2)	18(27.7)	65(26.4)
	Neutral Attitude (= 4, <5)	0(0.0)	3(60.0)	2(40.0)	5(2.0)
	Weak Negative Attitude (= 3, <4)	0(0.0)	1(50.0)	1(50.0)	2(0.8)
	Moderate Negative Attitude (= 2, <3)	0(0.0)	2(100.0)	0(0.0)	2(0.8)
	**Total**	**82(33.3)**	**82(33.3)**	**82(33.3)**	**246(100)**
Perceived norm	Very Positive (7)	2(66.7)	1(33.3)	0(0.0)	3(1.2)
	Positive (> = 6 <7)	38(36.2)	29(27.6)	38(36.2)	105(42.7)
	Moderately Positive (> = 5 <6)	31(40.3)	28(36.4)	18(23.4)	77(31.3)
	Moderately negative (> = 3 <4)	0(0.0)	6(37.5)	10(62.5)	16(6.5)
	Neither positive nor negative (> = 4 <5)	6(26.1)	11(47.8)	6(26.1)	23(9.3)
	Negative (> = 2 <3)	5(23.8)	6(28.6)	10(47.6)	21(8.5)
	Very negative (> = <2)	0(0.0)	1(100.0)	0(0.0)	1(0.4)
	**Total**	**82(33.3)**	**82(33.3)**	**82(33.3)**	**246(100)**
Behavioural control	Very high (7)	46(88.5)	3(5.8)	3(5.8)	52(21.1)
	High (> = 6 <7)	30(19.9)	66(43.7)	55(36.4)	151(61.4)
	Moderately high (> = 5 <6)	4(16.7)	6(25.0)	14(58.3)	24(9.8)
	Neither high nor low (> = 4 <5)	2(20.0)	3(30.0)	5(50.0)	10(4.1)
	Moderately low (> = 3 <4)	0(0.0)	0(0.0)	4(100.0)	4(1.6)
	Low (> = 2 <3)	0(0.0)	4(80.0)	1(20.0)	5(2.0)
	**Total**	**82(33.3)**	**82(33.3)**	**82(33.3)**	**246(100.0)**
Intention	Very high Intention	56(60.9)	18(19.6)	18(19.6)	92(37.4)
	High Intention	20(17.7)	52(46)	41(36.3)	113(45.9)
	Moderately high Intention	6(18.2)	8(24.2)	19(57.6)	33(13.4)
	Neither high nor low Intention	0(0.0)	4(50.0)	4(50.0)	8(3.3)
	**Total**	**82(33.3)**	**82(33.3)**	**82(33.3)**	**246(100.0)**

N(%): N is frequency, % is proportion. Proportions were arrived at by cross-tabulation of TPB constructs parameters and the study arms.

Client’s perceived risk of getting pregnant in early postpartum period was assessed by two parameters; perceived general risk and perceived individual risk of pregnancy in early postpartum period. The overall individual rating for perceived risk of getting pregnant in early postpartum period was arrived at by averaging the scores for responses for each of the questions thus very high perceived risk was average of 7, high perceived risk ≥6 and <7, moderately high perceived risk ≥5 and <6, neither high nor low high perceived risk ≥4 and <5, moderately low perceived risk ≥3 and <4, low perceived risk ≥2 and <3, and very low perceived risk ≥1 and <2. More than 85.0% of the participants had at least high perceived risk and the rest fell between low perceived risk to moderately high perceived risk with an average perceived risk of 6.1 (SD = 1.1).

Attitude towards behaviour was assessed using 4 Likert scale parameters targeting attitude towards uptake, attitude towards recommended minimum of 24 months of interbirth spacing, role of early PPFP in attaining recommended interbirth spacing and side effects of PPFP. Individual attitude towards PPFP was assessed using Likert scale indicators and overall rating was converted into a continuous variable by computing mean score of measurement parameters. These were summarized into frequency tables and measures of central tendency and dispersion. Overall attitude was converted into Likert scale as follows; very negative attitude (≥1, <2); moderate negative attitude (≥2, <3); weak negative attitude (≥3, <4); neutral attitude (≥4, <5); weak positive attitude (≥5, <6); moderate positive attitude (≥6, <7) and very positive attitude (≤7). The average rating of attitude towards early PPFP was high 5.8 (SD = 1.0). Average attitude was skewed towards positive with 96.4% of the attitude being rated in the positive spectrum as follows; moderate positive attitude 59.8%, weak positive attitude 26.4%, and very positive attitude 10.2%. The remaining 3.6% was accounted for by neutral attitude 2.0%, weak negative attitude 0.8% and moderate negative attitude 0.8%.

Perceived social normative beliefs was assessed as a precursor of intention which is a direct determinant of behaviour (early PPFP use). This was assessed by 5 Likert scale parameters on social normative beliefs on use of FP, early PPFP use, social pressure on use, partner approval and partner openness about early PPFP. Overall perceived social normative beliefs on PPFP were converted into a continuous variable by getting mean score of the Likert scale scores for each parameter. The mean of social normative beliefs was then calculated and used to classify perceived normative beliefs as follows; very positive (7), positive (≥6 <7), moderately positive (≥5 <6), neither positive nor negative (≥4 <5), moderately negative (≥3 <4), negative (≥2 <3), and very negative (≥ <2). Perceived normative beliefs about early PPFP were distributed across the whole spectrum from very negative perceived norm to very positive perceived norm. The average perceived norm was high 5.3 (SD = 1.3). The participants had a preponderance towards positive normative beliefs about early PPFP with 75.2% having either moderately positive (31.3%), positive (42.7%), or very positive (1.2%) perceived normative beliefs.

In this study, perceived individual control of PPFP choice was assessed by three (3) parameters geared towards confidence in using early PPFP, ease of PPFP use and ease of decision to use PPFP. Perceived individual control of PPFP choice was assessed using Likert scale questions and summarized into a continuous variable which was later converted into an ordinal scale. Before conversion to scale the findings of perceived individual control of PPFP choice were summarized into descriptive statistics for continuous variable. The mean of parameters of perceived individual control of early PPFP were calculated to estimate overall control beliefs thus classified as; very high (7), high (≥6 <7), moderately high (≥5 <6), neither high nor low (≥4 <5), moderately low (≥3 <4), low (≥2 <3), and very low (≥1 <2). The average perceived individual control of early PPFP was high 6.1 (SD = 0.9). Overall perceived behavioural control ranged from low (2%), moderately low (1.6%), neither high nor low (4.1%), moderately high (9.8%), high (61.4%) and very high (21.1%). The participants had a generally high level of perceived control of PPFP choice with 92.3% ranging in the moderately high, high and very high spectrum of perceived control.

Intention to use early PPFP was assessed using 3 Likert scale questions and summarized into a continuous variable which was later converted into an ordinal scale by averaging the score per participant. After getting the mean scores for intention the following criteria was used to classify it; very high intention (= 7), high intention (≥6, <7), moderately high intention (≥5, <6), neither high nor low intention (≥4, <5), moderately low intention (≥3, <4), low intention (≥2, <3), and very low intention (≥1, <2). The distribution of intention was as follows; neither high nor low intention 3.3%, moderately high intention 13.4%, high intention 45.9%, and very high intention 37.4% (M = 6.2, SD = 0.8).

### Structural Equation Modeling (SEM) for the adapted TPB

Theory of Planned Behaviour variable relationship with intention which is the key determinant of behaviour was assessed using structural equation modeling using SPSS AMOS. In addition to the traditional 3 constructs (behavioural control, attitude toward the behaviour and perceived normative beliefs about the behaviour) that predict behavioural intention, three more constructs were added to the model for this research. The added constructs were; client’s knowledge about PPFP, client satisfaction with PPFP counseling and client’s perceived risk of getting pregnant in the early postpartum period.

### Measurement model analysis

**Fig 3:**
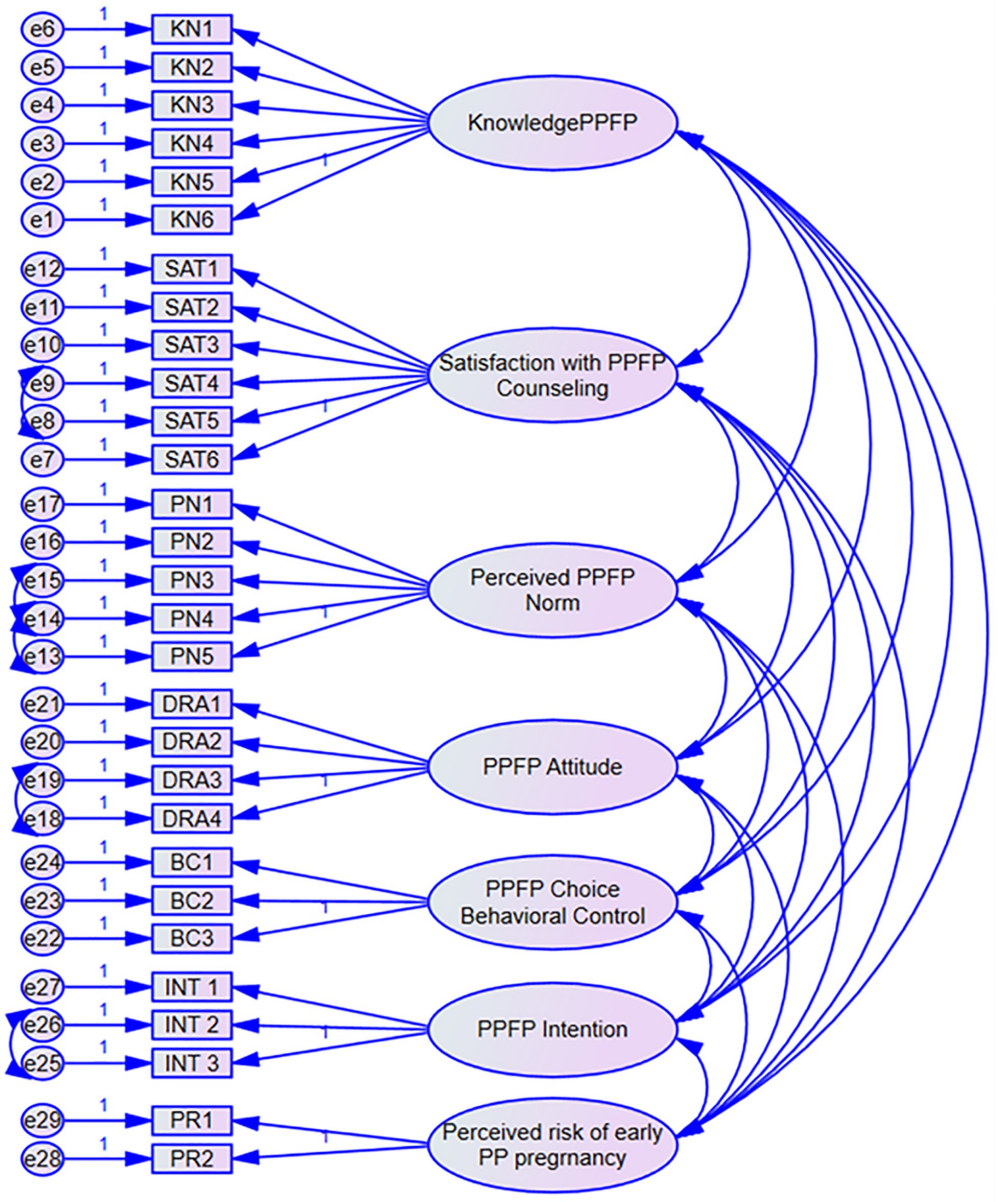
Measurement model. SPSS AMOS measurement modelling showing the relationships between latent variables and their observed indicators. Parameters for measurement of the constructs: KN—Knowledge about early PPFP; SAT—Satisfaction with PPFP Counseling; PN—Perceived Normative beliefs on early PPFP; DRA—Attitude towards PPFP; BC—Behaviour control of PPFP Choice; INT—PPFP Intention; PR—Perceived risk of early postpartum pregnancy; e- error terms.

Confirmatory Factor Analysis (CFA) model was designed (Fig 3), and computed using AMOS to test the measurement model. As part of confirmatory factor analysis, factor loadings were assessed for each item and the model modification indices proposed covariation of error terms (e) e8: e9, e13: e14, e14: e15, e18: e19 and e25: e26. This improved the model fit indices to acceptable thresholds.

### Model fit assessment

The model-fit indices were used to assess the model’s overall goodness of fit. Goodness of fit was assessed based on the prescribed thresholds for the C-Minimum Discrepancy Function by Degrees of Freedom (CMIN/df), Goodness of Fit Indices (GFI); the Tucker and Lewis (1973) Index (TLI); the Confirmatory Fit Index (CFI), [36,37,42]. Further assessment of fit was based on SPSS AMOS computed value of the Standardized Root Mean Square Residual (SRMR) and the Root Mean Square Error Approximation (RMSEA) [37,38]. The thresholds for the critical model indices were CMIN/df ≤3, GFI >0.9, CFI>0.9, TLI>0.9, SRMR<0.08 and RMSEA<0.08). The seven-construct modified TPB model yielded a good fit for the data: CMIN/df = 1.754, GFI = 0.857, CFI = 0.935, TLI = 0.925, SRMR = 0.0709, and RMSEA = 0.055. All the values were within their respective common acceptance levels [37].

#### Reliability and validity

Reliability was assessed by Cronbach’s Alpha and composite reliability and validity was assessed by Average Variance Extracted (AVE) as shown in Table 4. Construct reliability was assessed using Cronbach’s Alpha and composite reliability for indicators of each construct. Cronbach Alpha for each construct in the study was found to be above the required limited of 0.70 except for Knowledge of PPFP (0.688) [11,43,44]. Composite reliabilities were above threshold of 0.70 benchmark [40,42] except for perceived normative beliefs on early PPFP.

**Table 4:** Reliability of adapted TPB Model constructs parameters.

S. No.	Constructs in the Measurement model	Cronbach’s alpha >0.7 Valid	Composite reliability >0.7 Valid	Average Variance extracted >0.5 Valid
1	Knowledge about early PPFP	0.688	0.823	0.359
2	Satisfaction with PPFP Counseling	0.892	0.962	0.599
3	Perceived Normative beliefs on early PPFP	0.807	0.515	0.508
4	Attitude towards PPFP	0.844	0.758	0.621
5	Behaviour control of PPFP Choice	0.779	0.972	0.649
6	PPFP Intention	0.85	0.813	0.626
7	Perceived risk of early postpartum pregnancy	0.823	0.788	0.711

Reliability was assessed using Cronbach’s Alpha and composite reliability, and validity was assessed using Average Variance Extracted (AVE). Construct reliability was evaluated for each construct using both Cronbach’s Alpha and composite reliability. Cronbach Alpha values above 0.70 were considered acceptable, except for "Knowledge of PPFP" (0.688). Composite reliabilities were expected to be above the 0.70 threshold, except for perceived normative beliefs on early PPFP.

Convergent validity of scale items was estimated using Average Variance-Extracted (AVE). The Average Variance-Extracted values were above the threshold value of 0.50 [14,38,39]. Therefore, the scales used for each construct in the present study had the required convergent validity.

The measurement model demonstrates good overall reliability and validity. However, attention should be directed toward improving the internal consistency of the "Knowledge about early PPFP" construct.

### Structural model assessment

**Fig 4:**
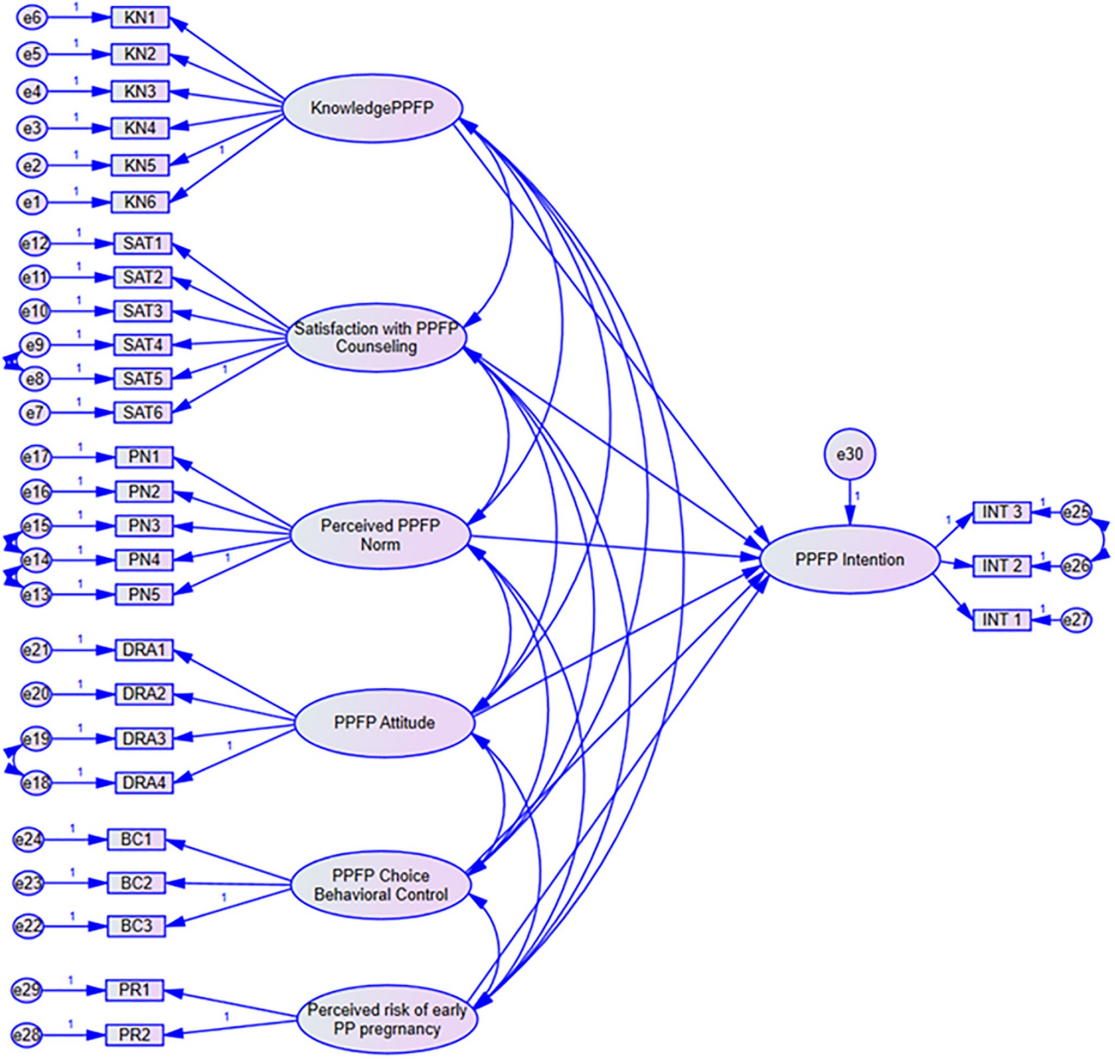
TPB structural model. SPSS AMOS Structural model showing the relationships/ paths between variables. Parameters for measurement of the constructs: KN—Knowledge about early PPFP; SAT—Satisfaction with PPFP Counseling; PN—Perceived Normative beliefs on early PPFP; DRA—Attitude towards PPFP; BC—Behaviour control of PPFP Choice; INT—PPFP Intention; PR—Perceived risk of early postpartum pregnancy; e- error terms.

A Structural Equation Model generated (Fig 4), through SPSS AMOS was used to test the relationships in the adapted TPB model. Goodness of fit was assessed based on the prescribed thresholds CMIN/df ≤3, GFI >0.9, CFI>0.9, TLI>0.9, SRMR<0.08 and RMSEA<0.08) [36,37,40].

**Step 0:** The model modification indices proposed covariation of error terms e8: e9, e13: e14, e14: e15, e18: e19 and e25: e26 from CFA. These model modifications were maintained in the Structural Equation Modeling (SEM).

**Fig 5:**
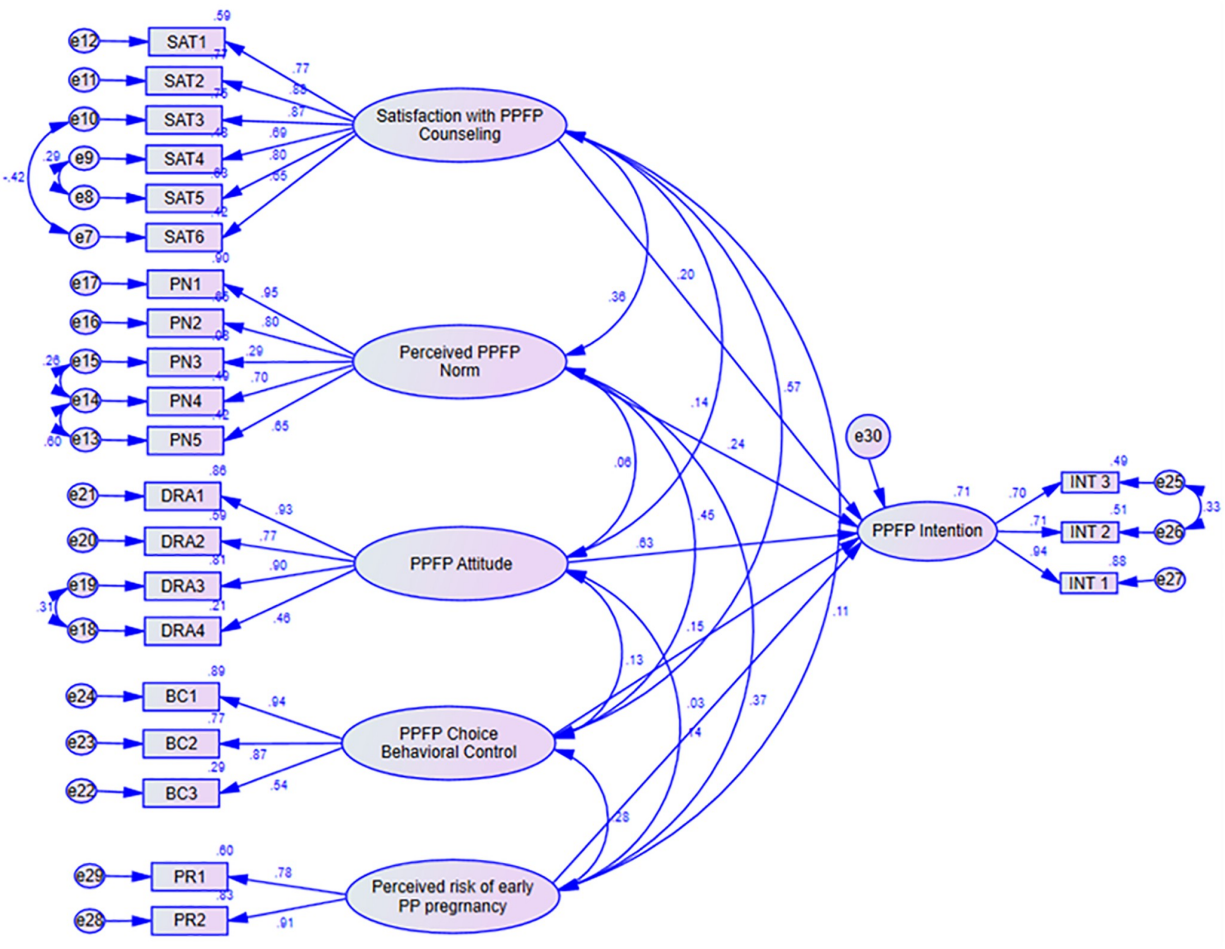
Optimized structural model. SPSS AMOS Model optimization/modification by; removing paths, covariances, or adjusting factor loadings. Knowledge for PPFP removed due to Multicollinearity. Parameters for measurement of the constructs: KN—Knowledge about early PPFP; SAT—Satisfaction with PPFP Counseling; PN—Perceived Normative beliefs on early PPFP; DRA—Attitude towards PPFP; BC—Behaviour control of PPFP Choice; INT—PPFP Intention; PR—Perceived risk of early postpartum pregnancy; e- error terms.

**Step 1**: Full structural model analysis focused on the effect of each construct on the other using path standard regression weights, validity of the indicators of each construct and correlation coefficients. It was then noted that knowledge had a low Cronbach’s alpha (0.688) and AVE (0.359) showing construct and convergence validity incoherence of its assessment criteria and indicators. Likewise, knowledge had the highest correlation with client satisfaction with counseling (0.816), PPFP behavioural control of choice (0.560) and perceived normative beliefs about early PPFP (0.352). The last 2 constructs which knowledge had high correlation with are core components of the TPB model. Considering these facts knowledge was removed from the initial model leading to the final structural model (Fig 5). This optimized the standardized regression weights of the other constructs that were fitted in the model. Thus, no further adjustments were made to the model. Model fit indices for the final model shown in table 96 fell within the acceptable range: CMIN/df = 1.874, GFI = 0.881, TLI = 0.937, CFI = 0.948, SRMR = 0.073, and RMSEA = 0.060.

#### Interaction of constructs in the final SEM model

Table 5 shows the interaction of constructs in the SEM model. The squared multiple correlation was 0.71 for intention to use PPFP in the first three months of delivery, this shows that 71% of variance in intention to use PPFP in the first three months of delivery was accounted for by the exogenous constructs in the model i.e. satisfaction PPFP counseling, perceived normative beliefs on early PPFP, attitude towards PPFP, behaviour control of PPFP choice, and perceived risk of early postpartum pregnancy.

**Table 5:** Interaction of constructs in the final SEM model.

S. No.	Hypothesized Relationship	Standardized regression estimates (β)	t-value	P-value
1	Satisfaction PPFP counseling	0.20	3.22	**0.001**
2	Perceived normative beliefs on early PPFP	0.24	3.98	**<0.0001**
3	Attitude towards PPFP	0.63	6.12	**<0.0001**
4	Behaviour control of PPFP choice	0.16	2.37	**0.018**
5	Perceived risk of early postpartum pregnancy	0.03	0.50	0.614
R^2^ for Intention to use PPFP in the early postpartum period 0.71				

Model output interpretation table with parameter estimates, standard errors, and model fit fit indices: CMIN/df = 1.874, GFI = 0.881, TLI = 0.937, CFI = 0.948, SRMR = 0.073, and RMSEA = 0.060.

All the constructs had a positive impact on intention to use PPFP in early postpartum period; satisfaction PPFP counseling (β = 0.197, t = 3.221, P = 0.001), perceived normative beliefs on early PPFP (β = 0.238, t = 3.981, P<0.0001), attitude towards PPFP (β = 0.632, t = 6.128, P<0.0001), behaviour control of PPFP choice (β = 0.157, t = 2.371, P = 0.018), and perceived risk of early postpartum pregnancy (β = .026, t = 0.506, P = 0.614).

### Moderation effect

Moderation effect of general aspects such as sociodemographic factors, process factors and quality of counseling, intimate partner relationship on the optimized model was assessed by ordinal logistic regression analysis as premeditated in the conceptualized model. It was not operationally feasible to model for each of these factors individually in the SEM phase. Likewise, now that the study had three distinct arms, the moderating effect of the intervention was assessed using ANOVA. These aspects were explored to enrich the interpretation of the results. Their effect was not directly modeled for in the SEM.

#### Analysis of moderation of general aspects

The moderation analysis for the general factors evaluated the influence of various factors on the intention for early postpartum family planning (PPFP). These factors were divided into different categories including sociodemographic characteristics of the clients, such as age, level of education, monthly income, marital status, and employment status. The pregnancy aspects analyzed included the number of antenatal care visits, the gestation period when PPFP counseling was offered, the presence of any comorbidities, the number of children, the health education received during pregnancy, and any complications that arose during pregnancy. The aspects related to labor, delivery, and postpartum were also considered, including health education received after birth, complications during labor and delivery, postpartum complications, and the health status of the woman three months postpartum. The previous family planning (FP) experience, including the rating of the woman’s previous experience with FP and the estimated cost of previous FP services, was taken into account, as well as the intimate partner relationship rated on a 7-point Likert scale ranging from very bad (1) to very good (7). Finally, the process-related aspects of the PPFP counseling, such as the waiting time for counseling, the speed of the counseling process, the mode of counseling, the quality of counseling, the fidelity to the process, and the willingness of the woman to set a postnatal appointment for PPFP, were also analyzed.

Ordinal logistic regression was used to demonstrate the moderating effect of general aspects; sociodemographic, pregnancy, labor and delivery, previous family planning experience, intimate partner relationship, and process-related aspects on the intention to use early postpartum family planning (PPFP) (Table 6). The sociodemographic characteristics fitted in the ordinal regression analysis model were; clients age, level of education, monthly income, marital status, and employment status were fitted in the ordinal regression analysis model. Only client’s age had a significant effect on intention to use early PPFP. An increase in age increased the odds of higher intention to use early PPFP (OR:1.1, 95% CI: 1.0–1.2, P = 0.022).

**Table 6:** Client and process aspects and intention to use early PPFP.

Category	Parameter	OR	95% CI	P-Value
Client related aspects	Clients Age	1.1	1.0–1.2	**0.022**
	Marital status	0.9	0.6–1.4	0.75
	Level of education	0.7	0.5–1.0	0.084
	Employment status	0.9	0.6–1.2	0.342
	Monthly income	0.9	0.5–1.5	0.58
	ANC visits number	0.9	0.8–1.1	0.647
	Gestation when PPFP counseling was done	1.0	0.9–1.1	0.255
	Existing illness	1.3	0.4–3.8	0.65
	Number of children	**1.3**	**1.1–1.6**	**0.004**
	Health education in pregnancy	**0.3**	**0.1–1.0**	**0.042**
	Complication during pregnancy	1.0	0.5–1.7	0.914
	Labour complications	0.7	0.3–1.5	0.384
	Postpartum complication	1.2	0.5–2.8	0.736
	Health education afterbirth	0.9	0.4–1.8	0.742
	Health status After pregnancy	0.9	0.3–2.6	0.861
	Health status in 3 months postpartum	0.5	0.2–1.3	0.151
	Rating previous experience with FP	1.4	-0.2–1.4	0.504
	Estimated cost of previous FP services	0.9	-0.6–1.0	0.895
	Intimate partner relationship	**1.5**	**1.2–1.8**	**<0.0001**
Process related aspects	FP counseling waiting time	1.0	0.8–1.1	0.097
	FP Counseling turnaround	**0.9**	**0.9–1.0**	**<0.0001**
	Mode of counseling	1.3	0.7–2.2	0.449
	Counseling Quality	1.0	1.0–1.1	0.105
	Fidelity to process	**2.6**	**1.9–3.6**	**<0.0001**
	Set Postnatal appointment	**2.4**	**1.3–4.6**	**0.007**

Ordinal regression analysis. OR- Odds Ratio, 95% CI– 95% confidence interval, Significance set at P≤0.05.

The pregnancy-related aspects fitted in the ordinal regression analysis model were; number of ANC visits, gestation when PPFP counselled, comorbidity, number of children, health education in pregnancy, and complication during pregnancy. Number of children and health education during pregnancy had significant effect on intentions for early PPFP. Having more children increased the odd of having high intention for early PPFP (OR:1.3; 95% CI: 1.1–1.6; P = 0.004). Being health educated was a binary variable with a 1 for “yes” and a 2 for “no” thus the tendency towards not having been educated in pregnancy reduced the odds of intending to use early PPFP (OR:0.3; 95% CI: 0.1–1.0; P = 0.042).

The labour, delivery and postpartum aspects fitted in the ordinal logistic regression model were; health education afterbirth, labour complications, postpartum complication, and health status in 3 months postpartum. None of the labour, delivery and postpartum related aspect had a significant effect on intentions for early PPFP. Rating of previous experience with FP and estimated cost of previous FP services did not elicit any significant effect on intentions for early PPFP.

Intimate partner relationship was rated on a 7-point Likert scale ranging from very bad intimate partner relationship (1) to very good intimate partner relationship (7). There was a significant relationship between intimate partner relationship and intentions for early PPFP. As the rating of intimate partner relationship increased, there was an increase in log odds of intentions for early PPFP (OR: 1.5; 95% CI: 1.2–1.8, P<0.0001).

Ordinal regression analysis to assess effects of process related aspects on intention to use early PPFP was done. FP counseling waiting time, counseling turnaround, mode of counseling, counseling quality, fidelity to process and accepting to set postnatal appointment for PPFP were fitted in the ordinal regression model. FP Counseling turnaround, fidelity to process and accepting to set postnatal appointment for early PPFP had a significant effect on intention to use early PPFP. As both fidelities to process and chance of setting postnatal appointment for early PPFP increased there was a congruent increase in the log odds of intention to use early PPFP (OR:2.62; 95% CI: 1.9–3.6, P<0.0001) and (OR:2.4; 95% CI:1.3–4.6, P = 0.007), respectively. The FP counseling turnaround had an inverse relationship with intention to use early PPFP whereby as counseling turnaround time increased the log odds of intention to use early PPFP reduced (OR:0.9; 95% CI: 0.9–1.0, P<0.0001). FP counseling waiting time had a similar relationship with intention as FP counseling turnaround time albeit not significant. Mode of counseling (individual counseling) and quality of counseling increased the odds of intention to use early PPFP although not significant.

*Moderating effect of intervention on intention for early PPFP per study arms*. A one-way ANOVA was done to assess the moderating effect of intervention on intention for early PPFP. A summary of descriptive statistics per arm was also done as a preamble to the main ANOVA test. Partial eta2 was used to estimate between arm effect size for ANOVA. The Nurses Arm had a mean intention for early PPFP score of 6.6 (SD = 0.9), the Community Arm had a mean score of 6.1 (SD = 0.9), and the Control Arm had a mean score of 6.0 (SD = 0.7). Across all study arms, the total mean intention score for Early PPFP was 6.2 (SD = 0.9).

A one-way ANOVA for intentions for early PPFP was done with Levene’s test showing that homogeneity of variance was met F(2,243) = 0.7, P = 0.490 thus Tukey’s post-hoc test used to estimate which arms had significant difference in intention, effect size estimation between arms. The ANOVA for intentions for early PPFP and study arms revealed a significant difference in the mean client intention scores between arms F(2,243) = 12.4, P<0.0001 with Tukey’s post-hoc test showing significantly higher intention for early PPFP between nurses’ arm and community arm (P<0.0001) and control arms (P<0.0001) with effect size varying for between arm comparisons. There was no significant difference in intention between the community arm and control arm (P = 0.986) as shown in Table 7.

**Table 7:** Analysis of variance (ANOVA) for difference in intentions for early PPFP.

Study Arm		MD	95% CI	P-value	Effect size
A	B	(A-B)			
Intervention	Control	0.3	0.1–0.5	0.012	0.03
Nurses	Community	0.5	0.2–0.8	<0.0001	0.09
Nurses	Control	0.6	0.3–0.9	<0.0001	0.11
Community	Control	0.1	-0.3–0.3	0.986	0.00

A and B are column labels, MD- Mean difference between A and B; 95% CI is the Confidence Interval for the Mean Difference (MD); Effect size was estimated by Partial eta^2^ (0.01 to <0.06—Small, 0.06 to <0.14 medium, ≥0.14 Large).

Tukey’s post hoc test was applied because homogeneity of variance was met.

Significance set at p≤0.05.

## Discussion

The current study assessed the effects of satisfaction PPFP counseling, perceived normative beliefs on early PPFP, attitude towards early PPFP, behavioural control of PPFP choice, and perceived risk of early postpartum pregnancy on intention for early PPFP uptake. The study demonstrates that satisfaction with PPFP counseling, perceived normative beliefs on early PPFP, attitude towards PPFP, and behaviour control of PPFP choice had a positive impact on intention to use PPFP in early postpartum period.

Satisfaction with postpartum family planning (PPFP) counseling can have a significant impact on a woman’s intention to start PPFP early such that a woman receiving comprehensive and personalized counseling is more likely to understand her options and feel confident in her ability to make informed decisions about her reproductive health. This, in turn, can increase her intention to start PPFP early, as she may feel more prepared to do so. A study in Ethiopia reports that women who received counseling were more likely to initiate PPFP within the first 6 weeks postpartum than those who did not receive counseling [45]. On the other hand, if a woman is not satisfied with the counseling she receives, she may be less likely to start PPFP early, as she may feel uncertain or uninformed about her options [46]. In the current study, the implementers of the intervention were given a refresher training on early postpartum family planning thus this is likely to have had an impact on intention for early PPFP.

Perceived normative beliefs on early PPFP can also affect a woman’s intention to start early. In the instance a woman perceives that it is socially acceptable to start PPFP immediately postpartum, she may be more likely to intend to do so A West-African study found that women who perceived that it was socially acceptable to start PPFP early were more likely to intend to do so than those who did not perceive it to be socially acceptable [47]. However, if she perceives that it is not socially acceptable to start PPFP early, she may be less likely to intend to do so. These perceived normative beliefs can be influenced by cultural and societal factors, as well as by the attitudes and behaviours of the people around her [48,49]. Attitude towards PPFP also plays an important role in a woman’s intention to start early. If a woman has a positive attitude towards PPFP, she may be more likely to intend to start early, as she may see the benefits of doing so Studies in India found that women who had a positive attitude towards PPFP were more likely to intend to start early than those who had a negative attitude [50,51]. Nevertheless, if she has a negative attitude towards PPFP, she may be less likely to intend to start early, as she may see it as burdensome or unnecessary. A woman’s attitude towards PPFP can be influenced by a variety of factors, including her personal beliefs, experiences, and knowledge about different PPFP methods [52]. Behaviour control also plays a role in a woman’s intention to start PPFP early. If a woman perceives that she has control over her ability to access and use PPFP, she may be more likely to intend to start early. A study in Bangladesh found that women who perceived that they had control over their ability to access and use PPFP were more likely to intend to start early than those who perceived that they did not have control [53]. Conversely, if she perceives that she does not have control over her ability to access and use PPFP, she may be less likely to intend to start early. Factors that can influence a woman’s perception of behaviour control include her socioeconomic status, access to healthcare, and cultural and societal barriers to accessing PPFP [14]. Perceived risk of early postpartum pregnancy can also influence a woman’s intention to start PPFP early. If a woman perceives that the risk of getting pregnant before she is ready is high, she may be more likely to intend to start PPFP early in order to prevent an unintended pregnancy. A study in Guatemala found that women who perceived a high risk of getting pregnant before they were ready were more likely to intend to start PPFP early than those who perceived a low risk [54]. However, if she perceives that the risk of getting pregnant is low, she may be less likely to intend to start PPFP early, as she may not see it as necessary. Factors that can influence a woman’s perception of the risk of early postpartum pregnancy include her personal experiences, knowledge about fertility, and access to accurate information about PPFP [55–57].

Older participants had significantly higher intention to use early PPFP thus suggesting that age is a factor in an individual’s desire or determination to use FP services and strategies during the early postpartum period. The finding that older participants had higher intention to use early PPFP may indicate that these individuals had a stronger motivation to prevent unintended pregnancy and were willing to take steps to do so. The relationship between age and intention to use early PPFP is likely complex as supported by several articles. Some of the reviewed previous research established that younger clients had higher intention for using PPFP as compared to their older counterparts [58,59]. The current study established that the number of children had a significant influence on the intention for early PPFP. Those who had more children had increased odds of intention to use PPFP. Having more children can increase the intention to use early postpartum family planning methods, as women may be more motivated to space or limit the number of their future pregnancies to ensure the well-being of their existing children and themselves, and also to prevent maternal morbidity and mortality [57]. That those who were not educated in pregnancy had lower intention to use early PPFP may indicate that these individuals had less knowledge about FP options and were therefore less motivated to prevent unintended pregnancy and take steps to do so. It is well established that education and access to information about pregnancy and reproductive health can have a significant impact on an individual’s intention to use early postpartum family planning (PPFP) methods. Studies have shown that women who lack access to education and information about pregnancy and reproductive health are less likely to have knowledge about and use early PPFP methods, such as intrauterine devices (IUDs) and hormonal contraceptives, which can prevent unintended pregnancies and improve maternal and child health outcomes. This is likely due to a lack of understanding about the benefits of early PPFP and concerns about side effects or potential complications. It is important to ensure that all individuals, especially those who may be at higher risk of unintended pregnancy, have access to education and information about PPFP in order to promote maternal and child health [57,60]. The finding that intimate partner relationship can influence intentions for early PPFP suggests that the relationship with one’s partner may be a factor in an individual’s desire or determination to use FP services and strategies during the early postpartum period. This may indicate that individuals with supportive partners are more likely to have a strong motivation to prevent unintended pregnancy and be willing to take steps to do so. On the other hand, individuals with less supportive partners may be less likely to have this motivation [61,62]. Research has shown that when PPFP services are provided with high fidelity, it can lead to increased intention to use early PPFP methods as is the case with the current study. This is because women are more likely to receive accurate and comprehensive information about the benefits and potential risks of these methods, and to have the opportunity to discuss their individual needs and preferences with a qualified provider. Fidelity to process in PPFP interventions is important as it increases the chances of women receiving accurate and comprehensive information about the benefits and potential risks of PPFP methods, as well as the opportunity to discuss their individual needs and preferences with a qualified provider, which in turn increases the intention to use early PPFP methods [62–64]. Setting postnatal appointment for early PPFP increased the intention for early PPFP shows that individuals who made plans to receive FP services in early postpartum period had a stronger motivation to prevent unintended pregnancy and were more willing to take steps to do so. The FP counseling turnaround had an inverse relationship with intention to use early PPFP whereby as counseling turnaround time increased the odds of intention to use early PPFP reduced. This denotes that the time it takes to receive counseling may be a factor in an individual’s desire or determination to use FP services and strategies during the early postpartum period so that individuals who received counseling in a shorter time period had a stronger motivation to prevent unintended pregnancy and were more willing to take steps to do so, while those who received counseling in a longer time period had a weaker motivation to do so. The FP counseling waiting time had a similar relationship with intention as FP counseling turnaround time albeit not significant. Mode of counseling (individual counseling) and quality of counseling increased the odds of intention to use early PPFP although not significant.

The results indicated that the nurses’ arm (where targeted antenatal FP counseling was provided by nurses) had a significant impact on intention for early PPFP compared to the control (routine antenatal care) and community arms (where counseling was provided by CHWs). These findings suggest that providing antenatal counseling on PPFP can positively influence expectant mothers’ intentions towards PPFP. Furthermore, the study highlights the critical role that nurses play in providing counseling and support to expectant mothers, which can have a significant impact on the uptake of PPFP. That the nurses in the study were more effective at providing information about PPFP to their clients during the postpartum period could be due to a variety of factors, such as the fact that the nurses in the study may have had more prior training and education about reproductive health and family planning, or that they had more opportunities to discuss these topics with their clients during the other ANC visits [65]. Likewise, the participants in the nurses arm may have had relatively different personal experiences that mediated their perceptions [60]. In a Tanzanian study, Keogh et al. (2015) found that including a 10-minute FP counseling session during ANC increased women’s intention to use FP but not their actual use of a method after birth [66]. Despite the lack of difference in mean intention scores between the community arm and the control arm in this study, it is still important to consider the potential role of community health workers in promoting early PPFP. Several studies have shown that Community health workers can provide valuable support and resources to new mothers in their own communities, and their involvement may be an effective means of increasing the uptake of early PPFP in other populations and settings [67–71].

### Study limitations

It is important to note that the findings of this study should be considered within the context of its limitations, such as the study setting and generalizability of the findings to other populations. The study is limited by selection bias, as it focuses solely on individuals attending antenatal care (ANC) clinics, potentially skewing the findings’ generalizability to the broader pregnant population. The study recognizes the need for a more diverse sample beyond ANC attendees to enhance external validity. Additionally, the study’s design limitation includes recruiting participants mostly in the third trimester, preventing the assessment of the intervention’s impact earlier in pregnancy and missing insights into factors influencing family planning decisions in the early stages.

## Conclusion and recommendations

In conclusion, the study found that satisfaction with postpartum family planning (PPFP) counseling, perceived normative beliefs, attitude towards PPFP, behavioural control of PPFP choice, and perceived risk of early postpartum pregnancy positively impact intention for early PPFP uptake. Notably, satisfaction with counseling, perceived normative beliefs, positive attitude, and behavioural control increased the intention to use PPFP early. Older participants and those with more children had higher intention, suggesting age and the number of children influence the desire for early PPFP. Education, partner relationship, and intervention fidelity were also associated with intention. The nurses’ arm had a significant impact, emphasizing the role of antenatal counseling by nurses in promoting early PPFP. However, the study has limitations, including selection bias focusing on ANC attendees and recruiting participants predominantly in the third trimester.

The study recommends prioritizing efforts to optimize antenatal FP counseling by well trained nurses to guarantee higher intention early PPFP. Additionally, interventions should address and promote positive perceived normative beliefs through community-level programs with culturally sensitive strategies. Fostering positive attitudes towards family planning and enhancing perceived behavioural control are key to reinforcing early PPFP intentions. Tailored interventions for younger individuals, support for those with more children, strengthening intimate partner relationships and maintaining fidelity to the process, are essential in guaranteeing higher intention for early PPFP. Lastly, it is recommended that future studies should employ a more inclusive participant recruitment strategy that goes beyond those exclusively engaged in antenatal care (ANC) and those in earlier trimesters of pregnancy.

## Supporting information

S1 Checklist CONSORT 2010 checklist of information to include when reporting a randomised trial*. (DOC)

S1 File (DOC)

## Abbreviations

ANC: Antenatal Care
ANOVA: Analysis of Variance
CHW: Community Health Worker
DMS: Director of Medical Services
FANC: Focused Antenatal Care
FP: Family Planning
IERC: Institutional Ethics Review Committee
KNBS: Kenya National Bureau of Statistics
MD: Mean Difference of intention between arms
MMUST: Masinde Muliro University of Science and Technology
NACOSTI: National Commission of Science, Technology and Innovation
PACTR: Pan African Clinical Trial Registry
PPFP: Postpartum Family Planning
RCT: Randomized Control Trial
SEM: Standard error of Mean
WHO: World Health Organization
